# Organ-Specific Toxicities Due to Radiation Therapy in Cancer Patients With or Without HIV Infection: A Systematic Review of the Literature

**DOI:** 10.3389/fonc.2018.00276

**Published:** 2018-07-30

**Authors:** Melody J. Xu, Alison Liewen, Luca Valle, Adam C. Olson, Nicola M. Zetola, Surbhi Grover

**Affiliations:** ^1^Department of Radiation Oncology, University of California, San Francisco, San Francisco, CA, United States; ^2^Department of Radiation Oncology, University of Pennsylvania, Philadelphia, PA, United States; ^3^Department of Radiation Oncology, University of California, Los Angeles, Los Angeles, CA, United States; ^4^Department of Radiation Oncology, Duke University Medical Center, Durham, NC, United States; ^5^Hubert Yeargan Center for Global Health, Duke University, Durham, NC, United States; ^6^Princess Marina Hospital, Gaborone, Botswana; ^7^Botswana-University of Pennsylvania Partnership, Gaborone, Botswana, Philadelphia, PA, United States

**Keywords:** human immunodeficiency virus, toxicity, radiotherapy, non-AIDS defining cancer, anal cancer, cervical cancer

## Abstract

**Background:** To synthesize published literature on the association between human immunodeficiency virus (HIV) infection and radiation therapy (RT)-related toxicities.

**Methods:** Two electronic databases, MEDLINE and Embase, were searched to identify studies published before November 2016 comparing RT-related toxicities between HIV-infected and HIV-uninfected patients receiving RT or chemoradiation therapy (CRT) for cancer. A qualitative synthesis of included articles and organ-specific toxicities was then performed.

**Results:** Of the 21 studies included in this review, 15 reported on anal cancer treatment, three on cervical cancer, two on Kaposi sarcoma, and one on prostate cancer. Reports in the pre-antiretroviral therapy (ART) or early ART era tended to identify increased morbidity and mortality with HIV infection. However, modern series incorporating more concurrent chemotherapy, conformal RT techniques, and ART administration result in fewer studies reporting toxicity differences in patients treated for anal and cervical cancers. When statistically significant, HIV-infected patients had higher rates of gastrointestinal toxicity with anal cancer CRT (up to 50%) and higher rates of hematologic toxicity with cervical cancer CRT (up to 31%). Of the 17 studies reporting treatment outcomes, nine suggest HIV-infected patients may have reduced local control and/or survival rates.

**Conclusions:** Overall, RT is likely similarly tolerated between HIV-infected and HIV-uninfected patients, especially with modern RT techniques. HIV-infected patients should continue to receive established standard of care RT and CRT dosing.

## Introduction

The global prevalence of cancer in people infected with human immunodeficiency virus (HIV) is increasing in parallel with the increased availability of cancer treatment worldwide. This has led to an increasing number of patients with HIV-associated malignancies receiving radiation therapy (RT) and chemoradiation therapy (CRT). Mortality and incidence of Acquired Immunodeficiency Syndrome (AIDS) defining cancers (ADCs) such as Kaposi sarcoma and non-Hodgkin lymphoma have decreased with expanding access to HIV antiretroviral treatment (ART) (1–3). However, non-AIDS-defining cancers (NADCs), such as anal cancer, prostate cancer, and Hodgkin lymphoma, have increased by three-fold from 1991 to 2005 (1, 2). In fact, there is now a higher rate of NADCs among HIV-infected patients compared to HIV-uninfected patients (4). The cause of this increase in NADCs among HIV-infected patients is likely multifactorial, including increased lifespan, lifestyle factors, oncogenic effects of viral co-infection, and chronic immune dysregulation resulting from HIV infection (5). Given the significant burden of malignancy among people living with HIV, and that approximately half of all cancer patients receive RT at some point during their disease course (6–8), determining the impact of HIV infection on RT-related toxicity is crucial.

Historically, concerns regarding the tolerability of RT in immunocompromised patients resulted in the exclusion of HIV-infected patients from most oncologic clinical trials. The relative paucity of clinical trial data has led to inconsistent reports regarding the association of HIV and toxicities arising from RT or CRT (9–11). A recent systematic review of studies reporting the efficacy and toxicity of RT in HIV-infected patients found that although increased toxicity was found in some studies, all cancers ought to be treated with standard-of-care regimens and there was no evidence to support treatment deintensification (12). We sought to further define the severity and type of organ-specific toxicity in HIV-infected patients treated with RT and CRT through systematic literature review.

## Methods

### Study design

A literature review protocol was drafted to include all elements of the Preferred Reporting Items for Systematic Review and Meta-Analyses (PRISMA) checklist (13). Retrospective cohort studies, prospective cohort studies, and case control studies directly comparing toxicities in HIV-infected vs. HIV-uninfected patients were eligible for this review. Case reports, case series, and cross-sectional prevalence studies were excluded due to lack of control groups. Only articles written in English were included.

### Participants, interventions, and comparators

The population under investigation was HIV-infected cancer patients. There were no restrictions based on age, gender, ethnicity, geographic location, co-morbidities, or number of patients. The interventions under investigation were RT or CRT. The primary outcome of interest was toxicity or other complications following RT. Acute toxicities were defined as those diagnosed within 6 months of initiating RT, and late toxicities were defined as those presenting 6 months after the initiation of RT. Secondary outcomes included local disease control, disease-free survival, overall survival, and hospitalization rates.

### Search strategy and data sources

The core strategy was reviewed prior to execution using the Peer Review for Electronic Search Strategies checklist (14). MEDLINE (via PubMed) and Embase were searched using the medical subject heading (MeSH) terms or equivalent keywords “radiotherapy,” “chemoradiotherapy,” “Antiretroviral Therapy, Highly Active,” “HIV,” “Acquired Immunodeficiency Syndrome,” “complications,” “adverse effects,” and “toxicity”. The searches were conducted on November 5, 2016 (Appendix 1). Reference lists of full-text articles were evaluated for additional unique titles not previously identified through database search.

### Study selection

Three researchers (AL, LV, AO) reviewed the search results. The final study selection process first included a review of titles followed by abstracts. When reviewing the titles, two of the three researchers independently reviewed the studies for inclusion, and each was classified as “include,” “unsure,” or “exclude”. If a study was classified “include” by either researcher, it was automatically advanced to the next level of review. If either reviewer classified a study as “unsure,” the third researcher made the final determination. If both researchers classified a study as “exclude,” it was excluded from further analysis. Reasons for exclusion were classified into one of the following groups: (1) Study: the study type was not a randomized control trial, retrospective or prospective cohort study containing both HIV-infected and HIV-uninfected cohorts, or case-control study (2) Population: the study population did not include cancer patients with HIV/AIDS, (3) Intervention: the population did not receive either RT or CRT, (4) Outcome: the study did not investigate toxicity outcomes, (5) Language: the study was not in English, or (6) Other.

When reviewing the abstracts, two of the three researchers independently reviewed the studies for inclusion using the same method as above. In order for studies to be included in the final data extraction stage, two researchers were required to be in agreement. Disagreements were resolved by discussion among all three researchers.

### Data extraction

After articles were selected for inclusion, data from each study was extracted using a structured form. Data extracted included study identification information, study design and methods, population characteristics (including HIV status, CD4 count, and type of cancer), treatments (including ART, RT, and CRT), outcome measures (organ-specific toxicity outcomes, disease-free survival, overall survival), results, author's key conclusions, and reviewer comments (Appendix 2). Data extraction focused on the following main endpoints of interest: discontinuation due to any adverse event; grade 3–4 adverse events; mortality; hospitalization; and gastrointestinal (GI), cutaneous, or respiratory adverse events. Each study's definition of these outcomes was also extracted. Study-specific toxicity definitions were recorded. Differences in data extraction were resolved by consensus among the three researchers.

Bias in each study was also assessed using the Cochrane Risk of Bias Tool. Specifically, studies were evaluated for selection bias, information bias, and bias in analysis. Risk of bias assessments were performed using the Cochrane Risk of Bias tool (15).

### Data analysis

Qualitative synthesis was conducted using evidence tables and written evidence summaries. Studies were summarized by cancer type to minimize heterogeneity in comparing treatment regimens and organ-specific toxicities. For each study, the study design and participant characteristics were noted. This included study size, cancer type and stage, HIV status, and initial CD4 counts or viral loads. Differences between HIV-infected and HIV-uninfected populations with regard to RT dosing and chemotherapy dosing (if applicable), RT duration, treatment compliance, toxicity, and survival outcomes were compared. HIV management was also examined, including use of ART. Due to small sample sizes and significant heterogeneity among studies, no meta-analyses were performed. Risk of bias was assessed for each individual study. The PRISMA reporting checklist can be found in Appendix 3.

## Results

### Search results

**Figure 1:**
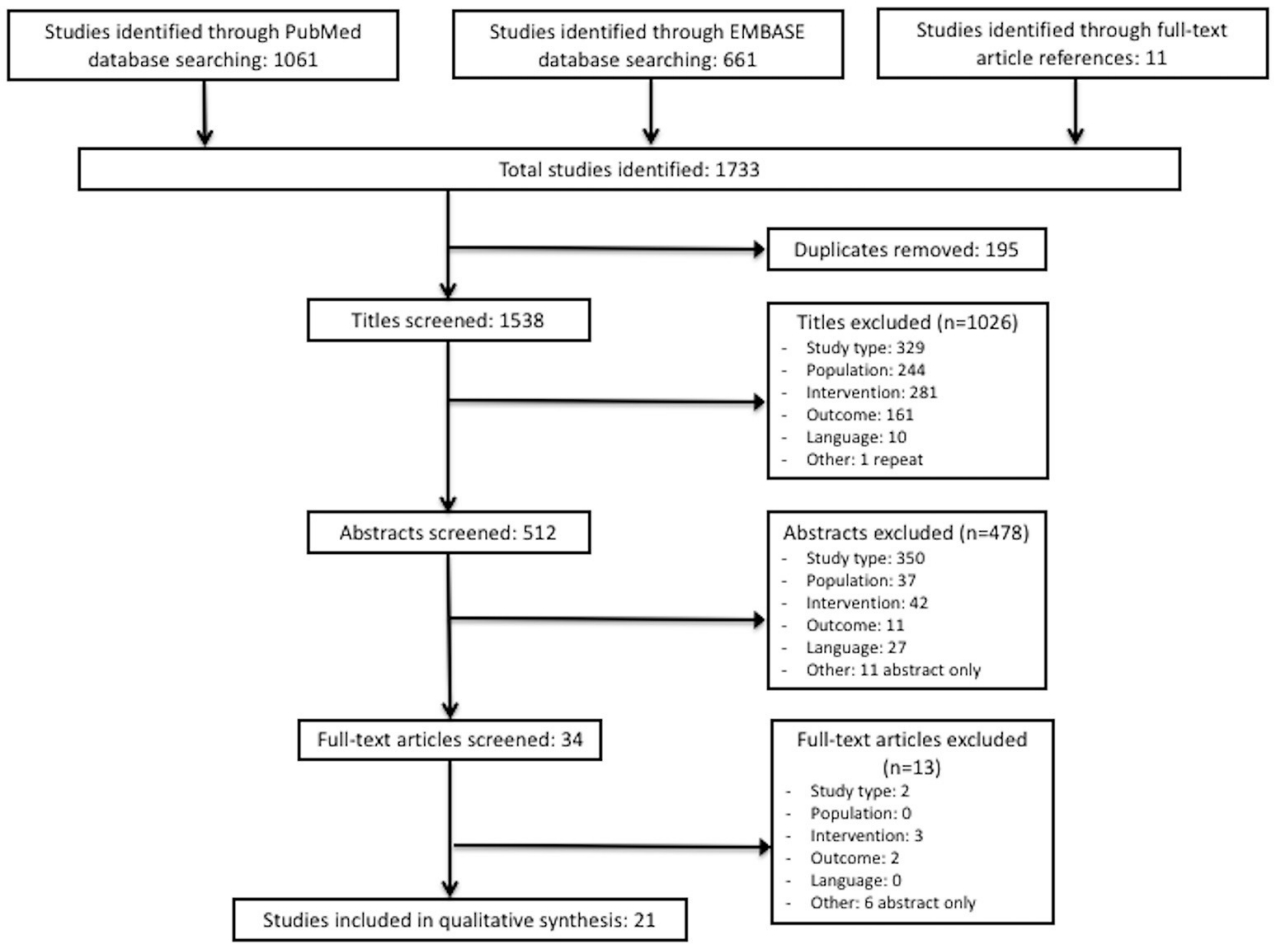
Consort diagram for study selection in this systematic review.

The search of electronic databases resulted in a total of 1,733 studies, including 1,061 studies from PubMed and 661 studies from Embase (Figure 1). Eleven unique studies were identified during full text article review and also assessed for inclusion in the review. After removing duplicates, a total of 1,538 unique studies remained. During the title review, 1,026 studies were excluded and 512 remained for abstract review. Reasons for exclusion based on title review were: incorrect type of study (*n* = 329), incorrect study population (*n* = 244), incorrect intervention (*n* = 281), incorrect outcome (*n* = 161), non-English language (*n* = 10), and duplicate (*n* = 1).

During the abstract review, 478 studies were excluded and 34 studies remained for full-text review. Reasons for exclusion based on abstract review were: incorrect type of study (*n* = 350), incorrect study population (*n* = 37), incorrect intervention (*n* = 42), incorrect outcome (*n* = 11), non-English language (*n* = 27), and abstract-only text (*n* = 11). During the full-text review, 13 studies were excluded, and 21 remained for the data extraction phase of this study (Appendix 4). Reasons for exclusion based on full-text review were: incorrect type of study (*n* = 2), incorrect intervention (*n* = 3), incorrect outcome (*n* = 2), and abstract-only text (*n* = 6) (Figure 1).

Of the 21 studies in the analysis, all were cohort studies including both HIV-infected and HIV-uninfected populations (16–36). Four were prospective studies and 17 were retrospective cohort studies. Altogether, these studies included a total of 2,320 patients, including 725 (31.3%) HIV-infected patients and 1,595 (68.8%) HIV-uninfected patients. Anal cancer was the most frequently studied disease site with 15 reports, followed by cervical cancer with three reports. All studies except Wieghard et al. (30) reported that HIV-infected cancer patients were more likely to be of younger age and in non-genitourinary (GU) cancers, all studies reported that HIV-infected cancer patients were more likely to be male. Seventeen studies originated from North America or Western Europe, and four studies originated from South Africa, Botswana, or Kenya. Toxicities were graded by either Radiation Therapy Oncology Group guidelines or Common Terminology Criteria for Adverse Events, both of which have similar criteria for severe grade 3 or higher toxicities. Five studies did not define a precise toxicity grading scheme (17, 22, 23, 26, 31). The most commonly assessed toxicities were acute skin, gastrointestinal, hematologic, and systemic (e.g., dehydration, pain) toxicities, which are listed for each study in Table 1.

**Table 1:** Studies reporting treatment toxicity in HIV-infected (HIV+) and HIV-uninfected (HIV–) patients treated for anal cancer in chronological publication order.

**Study n (HIV+, HIV–)**	**RT modality and dose**	**Reported treatment differences in HIV+**	**%Concurrent chemo**	**%ART**	**CD4 counts**	**Toxicities assessed**	**Significant toxicity differences in HIV+ vs. HIV–**
Holland et al. (23); *n =* 62 (7+, 55–)	3DCRT (53 Gy)	Chemo dose reduction (57%+ vs. 7%–) Increased treatment breaks (100%+ vs. 55%–) ^*^	100%+ 29% with 5FU/MMC 76% 65% with 5FU/MMC	ND	ND	Acute G3-4 skin, GI, heme, ID	Acute G3-4 toxicities including skin, GI, ID, pain, and dehydration (43%+ vs. 7%–)^*^
Kim et al. (24); *n =* 73 (13+, 60–)	ND	None	77%+ 5FU/MMC 70%– 5FU/MMC	ND	Median 146 (range 30–290)	Acute and late G3-4 skin, GI, heme	Increased acute G3-4 skin, GI, and heme toxicities (overall 80%+ vs. 30%–, *p <* 0.009)
Oehler-Janne et al. (27); *n =* 121 (40+, 81–)	3DCRT whole pelvis (45 Gy) + BT boost (14 Gy)	Increased duration of RT (*p =* 0.007) Fewer received chemo (55%+ vs. 72%–) ^*^ Inguinal RT more often (*p <* 0.005) and BT boost avoided (*p <* 0.0005)	55%+ with 30% 5FU/MMC 72%– with 64% 5FU/MMC	100%	Median 321 (range 2–1200)	Acute and late G3-4 skin, GI, heme	Increased acute G3-4 skin toxicities (35%+ vs. 17%–, *p =* 0.04) and decreased late G2 or higher proctitis (8%+ vs. 25%–, *p =* 0.03)
Abramowitz et al. (16); *n =* 151 (44+, 107–)	3DCRT whole pelvis + EBRT or BT boost (60–65 Gy)	More EBRT than BT (*p =* 0.04) and longer time between diagnosis and chemo (*p =* 0.016)	52%+ 83% with 5FU/cisplatin 56%– 70% with 5FU/cisplatin	98%	< 200 in 71%	Acute and late G3-4	None
Hogg et al. (22); *n =* 87 (21+, 66–)	ND	None	95%+ 5FU/MMC 89%– 5FU/MMC	95%	< 200 in 26%	Acute and late GI, GU, heme, ID, skin, systemic	Increased acute GI toxicities (48%+ vs. 24%–, *p =* 0.04)
Seo et al. (28); *n =* 36 (14+, 3 post-transplant, 19–)	3DCRT whole pelvis (43–68 Gy)	None	95%+ 5FU/MMC 100%– 5FU/MMC	71%	Mean 190	Acute and late skin, GI, GU	None
Fraunholz et al. (19); *n =* 70 (25+, 45–)	3DCRT whole pelvis (50.4 Gy) + EBRT or BT boost (5.4–10.8 Gy)	Fewer received full-dose chemo (72%+ vs. 91%–, *p =* 0.04)	72%+ 5FU/MMC 91%– 5FU/MMC	100%	Median 347.5 (range 63–930)	Acute G3-4 skin, GI, heme	None
Hammad et al. (21); *n =* 45 (13+, 32–)	EBRT whole pelvis (45–59 Gy in HIV+ and 45–63 Gy in HIV–)	Less likely to receive full dose chemo (reduced dose in 54%+ and 12%–)	85%+ 5FU/MMC 88%– 5FU/MMC	100%	Median 232 (range 125–460)	Acute G3-4 skin, heme, GI, brain, dehydration, ID	None
Munoz-Bongrand et al. (26); *n =* 46 (20+, 26–)	3DCRT whole pelvis + EBRT boost (60 Gy)	Increased duration of treatment due to acute toxicities (*p =* 0.027)	80%+ 5FU/cisplatin 73%– 5FU/cisplatin	95%	< 200 in 15% Median 306 (range 118–621)	Duration of CRT, acute toxicities	Increased duration of treatment due to acute toxicity (*p =* 0.027)
Doyen et al. (18); *n =* 105 (17+, 88–)	3DCRT whole pelvis (45 Gy) + EBRT boost (14.4 Gy) or BT boost (12 Gy in 3 fx)	None	71%+ 92% with 5FU/cisplatin 0% with 5FU/MMC 83%– 70% with 5FU/cisplatin 9% with 5FU/MMC	ND	ND	Acute and late G3-4 skin and GI	Increased late G3-4 GI toxicity (35.3%+ vs. 14.8%–, *p =* 0.04)
Amin et al. (17); *n =* 58 (11+ anal, 8+ lung, 39– anal)	Anal: 50.4 Gy Lung: 60–74 Gy	None	50% 5FU/MMC 36% other	100%	Mean 282	Acute heme	None
White et al. (29); *n =* 258 (53+, 205–)	EBRT (AP/PA or IMRT) + EBRT or BT boost (54 Gy, range 28–60 Gy)	None	98%+ 96% with 5FU/MMC 98%– 95% with 5FU/MMC	70%	Mean 455	Acute and late G3-4 skin, GI, heme	None
Grew et al. (20); *n =* 107 (39+, 68–)	3DCRT or IMRT whole pelvis (54 Gy)	Longer duration from biopsy to CRT (*p =* 0.042)	81%+ 5FU/MMC 88%– 5FU/MMC	ND	< 200 in 8% Median 381 (range 13–1177)	Acute fatigue, skin, GI	None
Wieghard et al. (30); *n =* 86 (14+, 72–)	EBRT (IMRT)	Less likely to receive MMC (*p =* 0.001)	36%+ 5FU/MMC 86%– 5FU/MMC	100%	Median 238	Treatment breaks, all G3-4 toxicities	None
Martin et al. (25); *n =* 142 (42+, 100–)	3DCRT or IMRT whole pelvis 50.4 Gy +/– EBRT boost (5.4–10.8 Gy)	More likely to receive chemo dose reduction, but not statistically significant	100%+ 5FU/MMC 100%– 5FU/MMC	ND	ND	Acute G3-4 skin, GI, heme, and pain	None

*Anterior-posterior and posterior-anterior parallel-opposed fields (AP/PA), anti-retroviral therapy (ART), brachytherapy (BT), chemotherapy (chemo), chemoradiotherapy (CRT), external beam radiation therapy (EBRT), fractions (fx), gastrointestinal (GI), genitourinary (GU), grade 3-4 (G3-4), infectious disease (ID), intensity-modulated radiation therapy (IMRT), mitomycin C (MMC), three-dimensional conformal radiation therapy (3DCRT), 5-fluorouracil (5FU), and not discussed (ND)*.

* *Study did not report p-values*.

All patients received RT, with concurrent chemotherapy (CRT) administered in 53–100% of anal cancer patients and in 0–84% of cervical cancer patients. RT was delivered as 3D conformal RT (3DCRT) or intensity modulated RT (IMRT). Where technique was not specified, Tables 1, 2 list “external beam RT (EBRT)” as the treatment technique. Boost RT doses were delivered with external beam or brachytherapy. Concurrent chemotherapy regimens are listed in Tables 1, 2. For anal cancer (Table 1), the majority of studies reported use of standard-of-care 5-fluorouracil (5FU) with mitomycin C (MMC) with a minority of studies reporting using of 5FU and cisplatin, which was an acceptable standard until the Radiation Therapy Oncology Group 9,811 results were reported in 2008 (37). For cervical cancer (Table 2), the preferred chemotherapy agent was cisplatin, which is also consistent with contemporary standards of care.

**Table 2:** Studies reporting treatment toxicity in HIV-infected (HIV+) and HIV-uninfected (HIV-) patients treated for cervical cancer.

**Study *n* (HIV+, HIV–)**	**RT modality and dose**	**Reported treatment differences in HIV+**	**%Concurrent chemo**	**%ART**	**CD4 counts**	**Toxicities assessed**	**Significant toxicity differences in HIV+ vs. HIV–**
Gichangi et al. (32); *n =* 218 (41+, 167–)	AP/PA using Co-60 (46.8 Gy, range 40–50 Gy)	More treatment interruptions (RR 2.3, *p =* 0.018)	0%	ND	ND	Acute G3-4 GU, GI, and skin	Higher acute G3-4 GU and overall toxicities: RR 4.8 (*p =* 0.002) and RR 6.7 (*p =* 0.003)
Simonds et al. (34); *n =* 213 (36+, 177–)	3DCRT (46–60 Gy) + BT boost (20–25 Gy in 4–5 fx)	More likely to receive RT alone (*p =* 0.01)	61%+ Cisplatin or carboplatin 76%– Cisplatin or carboplatin	100%	Median 341 (range 33–790) < 200 in 17%	Acute G3-4 overall, heme, renal, GI, GU, and skin	Increased acute G3-4 heme and overall toxicities: 30.6%+ vs. 10.2%– (*p =* 0.003) and OR 2.16 (CI 0.98–4.8; *p =* 0.05)
Dryden-Petersen et al. (31); *n =* 327 (231+, 96–)	EBRT (45–50 Gy) + BT boost (14–26 in 2–4 fx)	None	84%+ Cisplatin 73%– Cisplatin	82%	Median 397 (IQR 264–555)	Acute G3-4 overall, heme, renal, GI, GU, ID, skin	None

*Anterior-posterior and posterior-anterior parallel-opposed fields (AP/PA), anti-retroviral therapy (ART), brachytherapy (BT), chemotherapy (chemo), Cobalt 60 (Co-60), confidence interval (CI), fractions (fx), gastrointestinal (GI), genitourinary (GU), grade 3-4 (G3-4), interquartile range (IQR) odds ratio (OR), radiotherapy (RT), relative risk (RR), three-dimensional conformal radiation therapy (3DCRT), and not discussed (ND)*.

### Description of studies and qualitative synthesis by cancer type

#### Anal cancer

Fifteen of the total 21 studies examined anal cancer patients, totaling 1,439 patients, 373 of whom were HIV-infected (16–30). All of the studies were published between 2001 and 2017 (Table 1). Total RT dose administered and modalities were similar between studies, with 3DCRT as the most common method of EBRT and boost RT delivered through either EBRT or brachytherapy. In studies reporting differences in cancer treatment regimen by HIV status, HIV-infected patients were found to have more EBRT boost compared to brachytherapy (16, 24), higher rates of chemotherapy dose reduction(e.g., 72% HIV-infected patients received full dose chemotherapy vs. 91% HIV-uninfected patients) (19, 21), and longer delay between diagnosis and initiation of therapy(e.g., 84 days in HIV-infected vs. 54 days in HIV-uninfected) (20, 21). Of the 11 of the 15 anal studies reporting ART use (Table 1), 70–100% of HIV-infected patients were on ART at the time of treatment.

Ten of the 15 anal cancer studies found no difference in acute grade 3–4 toxicities in HIV-infected patients; only five reported increased toxicities (22–24, 26, 27). In two of those five studies conducted in the pre- or early ART era (1980–1990s), no information was available regarding the use of ART or viral control (23, 24). Therefore, although up to 80% of HIV-infected patients experienced acute grade 3–4 skin, GI, and hematologic toxicities (24) or required more treatment breaks and chemotherapy dose reduction (23), the conclusions are difficult to generalize to contemporary practice among HIV-infected patients on ART. The remaining three studies reported increased acute grade 3–4 toxicities in HIV-infected patients despite adequate HIV control. In these patients, ART was prescribed in 95–100% of patients and the median reported CD4 counts ranged from 306 to 321 cells per microliter, with < 30% of patients with CD4 counts < 200 (22, 26, 27). The incidence of acute toxicities in HIV-infected patients was primarily GI-related and double that of HIV-uninfected patients (17–24% in HIV-uninfected vs. 35–48% in HIV-infected), but not exceeding 50% in absolute incidence (22, 27). In one study, RT duration was found to be significantly longer in HIV-infected patients by 8 days (*p* = 0.007). Out of 1,439 patients, only one treatment-related death was reported in an HIV-infected patient and the death was due to bowel obstruction/perforation (28). Importantly, studies reported since 2014, which include over 600 patients, have not shown increased rates of acute toxicities in HIV-infected populations, increased RT duration, or increased rates of chemotherapy dose reduction.

Only two of the 15 anal cancer studies compared late toxicity rates; one study reported decreased late grade 2 or higher proctitis in HIV-infected patients 8% in HIV-infected vs. 25% in HIV-uninfected) (28) while another study found increased rates of late grade 3 to 4 ulcer formation (GI toxicity) among HIV-infected patients 35% in HIV-infected vs. 15% in HIV-uninfected) (18).

In the 13 post-ART anal cancer studies, seven evaluated toxicity, and clinical outcomes using CD4 counts or viral load stratification and found no differences in toxicity (20, 27, 28), colostomy rates (20, 22), local recurrences (20), distant metastases (20), or overall survival (16, 20, 22, 26, 28, 29).

#### Cervical cancer

Three studies examined RT toxicities in patients treated for cervical cancer (Table 2). Although two of three found increased rates of acute grade 3 or higher toxicity in the HIV-infected cohort (32, 34), the most recent and largest prospective cohort study to date did not (31). Types of acute toxicities varied by study, with the oldest study (32) identifying more GU toxicities using anterior-posterior and posterior-anterior (AP/PA) beam arrangements (relative risk, RR 4.8) and the more modern study utilizing 3DCRT, brachytherapy boost, and concurrent chemotherapy (34) finding more grade 3 or higher acute hematologic toxicities (31% in HIV-infected vs. 10% in HIV-uninfected) despite only 60% receiving concurrent chemotherapy. In the recent prospective cohort study by Dryden-Petersen et al. (31) reporting on the largest cohort of HIV-infected women to date with 231 patients, no differences in acute skin, GI, GU, hematologic, or renal toxicity were observed between HIV-infected and HIV-uninfected women.

#### Kaposi sarcoma

Two studies evaluated RT toxicities in treatment for Kaposi sarcoma. Chang et al. (17) compared toxicities and clinical responses between classic Kaposi sarcoma and epidemic Kaposi sarcoma. Patients were treated with RT alone from 1963 to 1990 using electron beam therapy, superficial x-rays, or Co-60 teletherapy. No grade 3–4 toxicities were found in either group. Stein et al. (31) also examined toxicities following RT in classic and epidemic Kaposi sarcoma in patients treated between 1978 and 1992. In this study, lesions were treated with electron beam therapy, superficial x-rays, or Co-60 teletherapy. Of the 15 patients with AIDS, ten were diagnosed with opportunistic infections, and the majority presented with disseminated skin involvement as part of advanced disease. Three patients developed grade 3 or higher toxicity, and all three were patients with AIDS-related Kaposi sarcoma of the oral mucosa. Because both studies were conducted in the pre-ART era, no data on CD4 count, or ART use was available.

#### Prostate cancer

One study examined RT toxicity in prostate cancer. Kahn et al. (33) matched each of 13 HIV-infected men with two HIV-uninfected control men to evaluate a total of 39 patients. Patients were matched by RT dose and treatment technique, tumor stage, Gleason score, prostate specific antigen, age, and race. The median CD4 count was 354 cells per microliter (range 50–1,002), and ART was administered in 62% of patients prior to diagnosis and in 69% of patients during treatment. The four-year biochemical failure rate was similar at 87–89%, but increased pre-treatment and post-treatment HIV viral loads were found to be significant risk factors for biochemical failure (*p* = 0.04). Acute GU and GI toxicities were significantly decreased in HIV-infected patients (*p* < 0.001 and *p* = 0.003, respectively), but only one HIV-uninfected patient (4%) experienced an acute grade 3 GU toxicity. Late GU and GI toxicities were also significantly decreased in HIV-infected patients (*p* < 0.001 and *p* < 0.001, respectively), with only one HIV-infected patient (7%) reporting a late grade 3 GU toxicity.

### Reported treatment outcomes

Although not the primary aim of this study, 17 of the 21 studies evaluated differences in clinical response to therapy by HIV-infection status in addition to cancer treatment-related toxicities (Table 3). Nine of the studies reported worse outcomes in HIV-infected compared to HIV-uninfected patients, either with poorer local control and recurrent rates (20, 22, 23, 27, 32) or worse survival (20, 23–26, 31).

**Table 3:** Studies reporting treatment outcomes in HIV-infected (HIV+) and HIV-uninfected (HIV–) patients.

**Study**	**Site**	**Outcomes evaluated**	**Significance**
Abramowitz et al. (16)	Anal	3-year LC	NS
		3-year OS	NS
		3-year relapse frequency	NS
Doyen et al. (18)	Anal	5-year cumulative colostomy incidence	NS
Dryden-Petersen et al. (31)	Cervix	Median OS	22 months HIV+ vs. 31 months HIV– (*p =* 0.02)
Fraunholz et al. (19)	Anal	Initial complete response	NS
		5-year LC	NS
		5-year OS	NS
Gichangi et al. (32)	Cervix	Risk of residual tumor at 4–7 months in HIV-infected	RR 3.7 (1.3–10.2), *p =* 0.009
Grew et al. (20)	Anal	3-year colostomy-free survival in HIV-	HR 3.23, *p =* 0.036
		3-year OS in HIV–	HR 2.33, *p =* 0.037
Hammad et al. (21)	Anal	Response duration	NS
		Median OS	NS
Hogg et al. (22)	Anal	Recurrence rate after 6 months	29% HIV+ vs. 8% HIV– (*p =* 0.009)
		Mean recurrence-free survival	30.6 months HIV+ vs. 45.3 months HIV– (*p =* 0.02)
		OS	NS
Holland et al. (23)	Anal	Time to failure ^*^	1.4 months HIV+ vs. 14.4 months HIV–
		Actuarial survival (2 years for HIV+, 4 years for HIV–)	29% HIV+ vs. 71% HIV– (*p <* 0.001)
Kahn et al. (33)	Prostate	Biochemical failure free survival	NS
		OS	NS
Kim et al. (24)	Anal	Initial complete response	NS
		Median cause-specific mortality	1.4 year HIV+ vs. 5.3 year HIV– (*p <* 0.05)
Martin et al. (25)	Anal	Initial complete response	NS
		5-year locoregional failure	NS
		5-year distant metastasis	NS
		5-year OS	NS
		5-year CSS	80.5% HIV+ vs. 93.8% HIV– (*p =* 0.03)
Munoz-Bongrand et al. (26)	Anal	5-year LC	NS
		5-year disease-free survival	NS
		5-year OS	39% HIV+ vs. 84% HIV– (*p =* 0.03)
Oehler-Janne et al. (27)	Anal	Initial complete response	NS
		5-year LC	38% HIV+ vs. 87% HIV– (*p =* 0.008)
		5-year relapse-free survival	35% HIV+ vs. 74% HIV– (*p =* 0.03)
		5-year OS	NS
		5-year CSS	NS
Seo et al. (28)	Anal	3-year colostomy-free survival	NS
		3-year OS	NS
		3-year CSS	NS
White et al. (29)	Anal	3-year colostomy-free survival	NS
		3-year progression-free survival	NS
		3-year OS	NS
		3-year CSS	NS
Wieghard et al. (30)	Anal	Initial complete response	NS
		Recurrence status	NS
		Colostomy-free survival	NS
		Median OS	NS

*Cause-specific survival (CSS), hazard ratio (HR), local control (LC), non-significant (NS), overall survival (OS), and relative risk (RR)*.

* *Study did not report p-value for this event*.

### Bias

Not every paper included details about CD4 count or ART administration. Among all studies that did, there was a wide range of CD4 values without obvious selection of only patients with well-controlled HIV infection. Reporting bias was found in all studies. In studies reporting changes in treatment technique for HIV-infected patients without associated increased toxicity, treatment selection bias favoring dose reduction or use of EBRT boost, rather than brachytherapy, may be present (16, 19, 21, 24, 30, 34). Observer bias may result in physicians grading toxicities differently based on a patient's known HIV status.

## Discussion

As patients with HIV live longer and develop NADCs, defining the risk of RT toxicity in HIV-infected patients is becoming increasingly relevant. In a systematic review of literature examining RT-related toxicity differences in HIV-infected and HIV-uninfected cohorts, we did not find compelling evidence that RT toxicity was increased in HIV-infected patients. However, several studies have reported increased acute grade 3 or higher toxicities in HIV-infected patients that may have the potential to negatively affect clinical outcomes. Thus, aggressive management of acute toxicities remains a high priority for HIV-infected patients.

Our systematic literature review identified 21 studies examining RT-induced toxicities. In studies conducted in the pre-ART or early ART era, where HIV-infected patients had overall poorer performance status, HIV-infected patients were observed to experience 2- to 6-fold increases in acute grade 3 or 4 skin, GI, pain, and hematologic toxicities that translated into increased treatment breaks and chemotherapy dose reductions (23, 24, 36). As a result of compromised treatment regimens, and, perhaps more importantly, reflecting the natural history of untreated HIV, both studies found that HIV-infected patients had significantly worse survival outcomes. However, in the post-ART era, severe RT-related toxicities among HIV-infected patients were found to be less prevalent.

In the 13 anal cancer studies conducted in the post-ART era, nine reported no differences in acute toxicity by HIV status. Of the four studies reporting increased toxicity rates (three reporting increased acute toxicity and one reporting increased late toxicity), the most common severe toxicity was GI-related (up to 48% of HIV-infected patients) followed by skin toxicity (up to 35%) in studies using 3DCRT techniques (18, 22, 27). Interestingly, in studies published after 2014 with greater proportions of patients treated using IMRT technique, acute skin, GI, and hematologic toxicity differences were not found. This could be due to a more favorable toxicity profile with IMRT compared to 3DCRT in anal cancer (38), perhaps with synergistic effects of better and earlier HIV treatment, but additional studies are required to further explore this association.

Only three studies evaluating cervical cancer treatment were identified. With increasing use of ART, increased concurrent chemotherapy administration, and improvements in technique from AP/PA to 3DCRT, toxicity profiles have changed from primarily GU-related to hematologic-related (32, 34), with the most recent large prospective cohort study finding no differences in severe toxicities. These findings support the notion that increasingly conformal RT delivery techniques can improve acute toxicities, even in the context of concurrent chemotherapy in cervical cancer (38).

Grade 3 or higher skin toxicities were rare among Kaposi sarcoma studies. However, Stein et al. (36) reported more opportunistic infections and disseminated skin involvement in HIV-infected patients and found that all three of their patients who experienced grade 3 toxicities were HIV-infected. These observations likely reflect the challenges in treating patients with advanced AIDS in the pre-ART era rather than RT-related skin toxicities, but modern reports comparing toxicity in patients with HIV treated with superficial RT for skin cancers are needed.

Lastly, one study examined prostate cancer treatments and found that HIV-infected patients were less likely to experience acute and late GU and GI toxicities (33). Unfortunately, the reasons for decreased toxicities in HIV-infected patients were not determined. However, grade 3 toxicities were rare as only one HIV-uninfected patient experienced an acute grade 3 toxicity and only one HIV-infected patient experienced a late grade 3 GU toxicity. Altogether, this study appears to support the evidence that no significant differences in severe toxicities are observed between HIV-infected and HIV-uninfected patients.

Of the 17 studies commenting on differences in treatment outcomes, nine reported either worse local disease control or survival. Some explanations for these outcome disparities included compromised treatment regimens due to acute toxicities (24, 26, 32) and differences in biology, such as early oncologic progression (31), HIV-related comorbidities or persistence of the human papilloma virus implicated in anal and cervical cancers (20, 22). Further studies are needed to better define the contribution of biology and compromised treatment regimens to clinical outcomes in HIV-infected patients.

Many studies attempted to stratify toxicity and clinical outcomes by HIV control. CD4 counts, viral load, and ART administration did not appear to strongly affect toxicity or clinical outcomes in any anal or cervical cancer studies evaluating this effect. In the single prostate cancer study, increased pre- and post-RT viral load was associated with increased risk of biochemical failure, but decreased CD4 count was not (33). However, the results from these studies should be interpreted with caution due to small sample size and inconsistent ART initiation times. For example, although Simonds et al. (34) report 100% ART administration, only 44% of the patients were on ART prior to RT, and the remaining patients started ART during or after RT. Other studies with higher percentages of HIV-infected patients initiated on ART prior to RT may not have described the duration of ART, HIV control, or specific components of ART administered (22, 23, 33). There remains heterogeneity in using CD4 counts, viral load, and ART to measure HIV control, and future studies evaluating the effect of HIV control on toxicity and clinical outcomes are still greatly needed.

Many of the studies in this review were limited by their small sample sizes and retrospective study design. Statistical comparisons of toxicities were frequently limited to acute toxicities alone; chronic toxicities were often difficult to assess due to limited follow-up. Although these studies encompass patients in both developing and developed countries, the vast majority originated from academic centers in the US and Europe and may therefore not be generalizable to other countries. Two studies had significant loss to follow-up (32, 36). Five studies did not define a precise toxicity grading scheme (17, 22, 23, 26, 31). Potential biases included treatment selection bias and observer bias, where physicians may prescribe treatment regimens and grade toxicities differently depending on knowledge of a patient's HIV status.

In summary, the results of this systematic review suggest that RT is likely similarly tolerated by HIV-infected and HIV-uninfected patients, especially in the era of ART, conformal RT techniques, even when combined with chemotherapy. In studies where HIV-infected patients experienced increased grade 3 or 4 acute toxicities, toxicities were primarily GI for anal cancer patients or hematologic for cervical cancer patients, resulting in treatment delays or chemotherapy dose reductions that may have a negative impact on cancer control outcomes. Because there is no definitive evidence that HIV-infected patients are more likely to experience acute toxicities, especially in the era of modern ART, and the available data argues against upfront dose reduction or treatment de-intensification due to the negative impact on clinical outcomes, we recommend continuing with established standard of care dosing with aggressive treatment for acute toxicities. Additional studies with larger comparative cohorts treated in the modern ART era with advanced RT techniques, focused on different cancer sites, and more homogeneous cancer treatment regimens are needed to strengthen the evidence on this topic.

## Author contributions

The study design was conceptualized by MX, AL, and SG, with data collection performed by AL, LV, and AO. Data analysis was performed by MX, AL, LV, and SG. Manuscript writing and editing was performed by MX, NZ, and SG.

### Conflict of interest statement

The authors declare that the research was conducted in the absence of any commercial or financial relationships that could be construed as a potential conflict of interest.
