# Supplementary material for: Organ-Specific Toxicities Due to Radiation Therapy in Cancer Patients With or Without HIV Infection: A Systematic Review of the Literature
Source: Front Oncol. 2018 Jul 30;8:276. doi: 10.3389/fonc.2018.00276 (PMC6077254; doi:10.3389/fonc.2018.00276)
Supplement: Supplementary file 1 [file Data_Sheet_1.DOCX]

**Appendix 1. MEDLINE/PubMed and Embase search terms.**

**MEDLINE/PubMed**:

11/5/16 Search ((((((((AIDS) OR HIV) OR acquired immunodeficiency syndrome) OR human immunodeficiency virus)) AND ((radiation therapy) OR chemoradiation therapy)) AND (((toxicity) OR adverse effects) OR complications))) 1061

DETAILS OF SEARCH: (((((("acquired immunodeficiency syndrome"[MeSH Terms] OR ("acquired"[All Fields] AND "immunodeficiency"[All Fields] AND "syndrome"[All Fields]) OR "acquired immunodeficiency syndrome"[All Fields] OR "aids"[All Fields]) OR ("hiv"[MeSH Terms] OR "hiv"[All Fields])) OR ("acquired immunodeficiency syndrome"[MeSH Terms] OR ("acquired"[All Fields] AND "immunodeficiency"[All Fields] AND "syndrome"[All Fields]) OR "acquired immunodeficiency syndrome"[All Fields])) OR ("hiv"[MeSH Terms] OR "hiv"[All Fields] OR ("human"[All Fields] AND "immunodeficiency"[All Fields] AND "virus"[All Fields]) OR "human immunodeficiency virus"[All Fields])) AND (("radiotherapy"[Subheading] OR "radiotherapy"[All Fields] OR ("radiation"[All Fields] AND "therapy"[All Fields]) OR "radiation therapy"[All Fields] OR "radiotherapy"[MeSH Terms] OR ("radiation"[All Fields] AND "therapy"[All Fields]) OR "radiation therapy"[All Fields]) OR (("chemoradiotherapy"[MeSH Terms] OR "chemoradiotherapy"[All Fields] OR "chemoradiation"[All Fields]) AND ("therapy"[Subheading] OR "therapy"[All Fields] OR "therapeutics"[MeSH Terms] OR "therapeutics"[All Fields])))) AND ((("toxicity"[Subheading] OR "toxicity"[All Fields]) OR ("adverse effects"[Subheading] OR ("adverse"[All Fields] AND "effects"[All Fields]) OR "adverse effects"[All Fields])) OR ("complications"[Subheading] OR "complications"[All Fields])))

**Embase**:

11/5/16 Search 1: radiotherapy OR chemoradiotherapy AND human immunodeficiency virus OR acquired immune deficiency syndrome AND toxicity OR adverse effects OR complications

Search Results: 661

**Appendix 2. Data extraction form.** RT = radiotherapy; CRT = chemoradiotherapy; RR = relative risk; OR = odds ratio; and HR = hazard ratio.

| **Full study data extraction** | |
| --- | --- |
| Study identification |  |
| Study design: retrospective cohort, prospective cohort, or case control |  |
| Participant characteristics: study size, geography, number of HIV-infected vs. HIV-uninfected, CD4 count, cancer type, population subgroups |  |
| Exposure: RT, CRT |  |
| Outcome: toxicity, disease control, overall survival |  |
| Results: p-value, RR, OR, HR |  |
| Discussion: key conclusions, reviewer comments |  |
| Bias: treatment compliance, clinical outcomes, HIV management |  |

**Appendix 3. PRISMA Checklist.**

| **Section/topic** | **#** | **Checklist item** | **Reported on page #** |  |
| --- | --- | --- | --- | --- |
| **TITLE** | | |  |  |
| Title | 1 | Identify the report as a systematic review, meta-analysis, or both. | Title |  |
| **ABSTRACT** | | |  |  |
| Structured summary | 2 | Provide a structured summary including, as applicable: background; objectives; data sources; study eligibility criteria, participants, and interventions; study appraisal and synthesis methods; results; limitations; conclusions and implications of key findings; systematic review registration number. | 1 |  |
| **INTRODUCTION** | | |  |  |
| Rationale | 3 | Describe the rationale for the review in the context of what is already known. | 2 |  |
| Objectives | 4 | Provide an explicit statement of questions being addressed with reference to participants, interventions, comparisons, outcomes, and study design (PICOS). | 2 |  |
| **METHODS** | | |  |  |
| Protocol and registration | 5 | Indicate if a review protocol exists, if and where it can be accessed (e.g., Web address), and, if available, provide registration information including registration number. | n/a |  |
| Eligibility criteria | 6 | Specify study characteristics (e.g., PICOS, length of follow-up) and report characteristics (e.g., years considered, language, publication status) used as criteria for eligibility, giving rationale. | 3 |  |
| Information sources | 7 | Describe all information sources (e.g., databases with dates of coverage, contact with study authors to identify additional studies) in the search and date last searched. | 3 |  |
| Search | 8 | Present full electronic search strategy for at least one database, including any limits used, such that it could be repeated. | 3 |  |
| Study selection | 9 | State the process for selecting studies (i.e., screening, eligibility, included in systematic review, and, if applicable, included in the meta-analysis). | 4 |  |
| Data collection process | 10 | Describe the method of data extraction from reports (e.g., piloted forms, independently, in duplicate) and any processes for obtaining and confirming data from investigators. | 4-5 |  |
| Data items | 11 | List and define all variables for which data were sought (e.g., PICOS, funding sources) and any assumptions and simplifications made. | 5, Appendix 2 |  |
| Risk of bias in individual studies | 12 | Describe methods used for assessing risk of bias of individual studies (including specification of whether this was done at the study or outcome level), and how this information is to be used in any data synthesis. | 6, Appendix 2 |  |
| Summary measures | 13 | State the principal summary measures (e.g., risk ratio, difference in means). | n/a |  |
| Synthesis of results | 14 | Describe the methods of handling data and combining results of studies, if done, including measures of consistency (e.g., I^2^) for each meta-analysis. | 5 |  |
| Risk of bias across studies | 15 | Specify any assessment of risk of bias that may affect the cumulative evidence (e.g., publication bias, selective reporting within studies). | 6, Appendix 2 |  |
| Additional analyses | 16 | Describe methods of additional analyses (e.g., sensitivity or subgroup analyses, meta-regression), if done, indicating which were pre-specified. | n/a |  |
| **RESULTS** | | |  | |
| Study selection | 17 | Give numbers of studies screened, assessed for eligibility, and included in the review, with reasons for exclusions at each stage, ideally with a flow diagram. | 6 | |
| Study characteristics | 18 | For each study, present characteristics for which data were extracted (e.g., study size, PICOS, follow-up period) and provide the citations. | 7-10, Tables 1-3 | |
| Risk of bias within studies | 19 | Present data on risk of bias of each study and, if available, any outcome level assessment (see item 12). | 11 | |
| Results of individual studies | 20 | For all outcomes considered (benefits or harms), present, for each study: (a) simple summary data for each intervention group, and (b) effect estimates and confidence intervals, ideally with a forest plot. | 7-10, Tables 1-3 | |
| Synthesis of results | 21 | Present the main results of the review. If meta-analyses are done, include for each, confidence intervals and measures of consistency. | 7-10, Tables 1-3 | |
| Risk of bias across studies | 22 | Present results of any assessment of risk of bias across studies (see Item 15). | 11 | |
| Additional analysis | 23 | Give results of additional analyses, if done (e.g., sensitivity or subgroup analyses, meta-regression [see Item 16]). | n/a | |
| **DISCUSSION** | | |  | |
| Summary of evidence | 24 | Summarize the main findings including the strength of evidence for each main outcome; consider their relevance to key groups (e.g., healthcare providers, users, and policy makers). | 12-14 | |
| Limitations | 25 | Discuss limitations at study and outcome level (e.g., risk of bias), and at review-level (e.g., incomplete retrieval of identified research, reporting bias). | 14 | |
| Conclusions | 26 | Provide a general interpretation of the results in the context of other evidence, and implications for future research. | 14-15 | |
| **FUNDING** | | |  | |
| Funding | 27 | Describe sources of funding for the systematic review and other support (e.g., supply of data); role of funders for the systematic review. | Title | |

**Appendix 4. List of articles included in the systematic review.**

Abramowitz L, Mathieu N, Roudot-Thoraval F, Lemarchand N, Bauer P, Hennequin C, et al. Epidermoid anal cancer prognosis comparison among HIV+ and HIV- patients. Aliment Pharmacol Ther. 2009;30(4):414-21.

Amin RM, Ebisutani KM, Zeng J. Impact of HIV Status and Protease Inhibitor Use on Blood Counts during Chemotherapy and Radiation Treatment for Anal and Lung Cancers. J J Rad Oncol. 2016;3(1): 025.

Chang LF, Reddy S, Shidnia H. Comparison of radiation therapy of classic and epidemic Kaposi's sarcoma. Am J Clin Oncol. 1992;15(3):200-6.

Doyen J, Benezery K, Follana P, Ortholan C, Gérard JP, Hannoun-Levi JM, et al. Predictive factors for early and late local toxicities in anal cancer treated by radiotherapy in combination with or without chemotherapy. Dis Colon Rectum. 2013;56(10):1125-33.

Dryden-Peterson S, Bvochora-Nsingo M, Suneja G, Efstathiou JA, Grover S, Chiyapo S, et al. HIV Infection and Survival Among Women With Cervical Cancer. J Clin Oncol. 2016;34(31):3749-57.

Fraunholz I, Rabeneck D, Gerstein J, Jäck K, Haberl A, Weiss C, et al. Concurrent chemoradiotherapy with 5-fluorouracil and mitomycin C for anal carcinoma: are there differences between HIV-positive and HIV-negative patients in the era of highly active antiretroviral therapy? Radiother Oncol. 2011;98(1):99-104.

Gichangi P, Bwayo J, Estambale B, Rogo K, Njuguna E, Ojwang S, et al. HIV impact on acute morbidity and pelvic tumor control following radiotherapy for cervical cancer. Gynecol Oncol. 2006;100(2):405-11.

Grew D, Bitterman D, Leichman CG, Leichman L, Sanfilippo N, Moore HG, et al. HIV Infection Is Associated With Poor Outcomes for Patients With Anal Cancer in the Highly Active Antiretroviral Therapy Era. Dis Colon Rectum. 2015;58(12):1130-6.

Hammad N, Heilbrun LK, Gupta S, Tageja N, Philip PA, Shields AF, et al. Squamous cell cancer of the anal canal in HIV-infected patients receiving highly active antiretroviral therapy: a single institution experience. Am J Clin Oncol. 2011;34(2):135-9.

Hogg ME, Popowich DA, Wang EC, Kiel KD, Stryker SJ, Halverson AL. HIV and anal cancer outcomes: a single institution's experience. Dis Colon Rectum. 2009;52(5):891-7.

Holland JM, Swift PS. Tolerance of patients with human immunodeficiency virus and anal carcinoma to treatment with combined chemotherapy and radiation therapy. Radiology. 1994;193(1):251-4.

Kahn S, Jani A, Edelman S, Rossi P, Godette K, Landry J, et al. Matched cohort analysis of outcomes of definitive radiotherapy for prostate cancer in human immunodeficiency virus-positive patients. Int J Radiat Oncol Biol Phys. 2012;83(1):16-21.

Kim JH, Sarani B, Orkin BA, Young HA, White J, Tannebaum I, et al. HIV-positive patients with anal carcinoma have poorer treatment tolerance and outcome than HIV-negative patients. Dis Colon Rectum. 2001;44(10):1496-502.

Martin D, Balermpas P, Fokas E, Rödel C, Yildirim M. Are there HIV-specific Differences for Anal Cancer Patients Treated with Standard Chemoradiotherapy in the Era of Combined Antiretroviral Therapy? Clin Oncol (R Coll Radiol). 2017;29(4):248-55.

Munoz-Bongrand N, Poghosyan T, Zohar S, Gerard L, Chirica M, Quero L, et al. Anal carcinoma in HIV-infected patients in the era of antiretroviral therapy: a comparative study. Dis Colon Rectum. 2011;54(6):729-35.

Oehler-Jänne C, Huguet F, Provencher S, Seifert B, Negretti L, Riener MO, et al. HIV-specific differences in outcome of squamous cell carcinoma of the anal canal: a multicentric cohort study of HIV-positive patients receiving highly active antiretroviral therapy. J Clin Oncol. 2008;26(15):2550-7.

Seo Y, Kinsella MT, Reynolds HL, Chipman G, Remick SC, Kinsella TJ. Outcomes of chemoradiotherapy with 5-Fluorouracil and mitomycin C for anal cancer in immunocompetent versus immunodeficient patients. Int J Radiat Oncol Biol Phys. 2009;75(1):143-9.

Simonds HM, Neugut AI, Jacobson JS. HIV Status and Acute Hematologic Toxicity Among Patients With Cervix Cancer Undergoing Radical Chemoradiation. Int J Gynecol Cancer. 2015;25(5):884-90.

Stein ME, Lakier R, Spencer D, Dale J, Kuten A, MacPhail P, et al. Radiation therapy for non-AIDS associated (classic and endemic African) and epidemic Kaposi's sarcoma. Int J Radiat Oncol Biol Phys. 1994;28(3):613-9.

White EC, Khodayari B, Erickson KT, Lien WW, Hwang-Graziano J, Rao AR. Comparison of Toxicity and Treatment Outcomes in HIV-positive Versus HIV-negative Patients With Squamous Cell Carcinoma of the Anal Canal. Am J Clin Oncol. 2017;40(4):386-92.

Wieghard N, Hart KD, Kelley K, Lu KC, Herzig DO, Mitin T, et al. HIV positivity and anal cancer outcomes: A single-center experience. Am J Surg. 2016;211(5):886-93.
